# First person – Helen Eachus

**DOI:** 10.1242/dmm.050452

**Published:** 2023-10-02

## Abstract

First Person is a series of interviews with the first authors of a selection of papers published in Disease Models & Mechanisms, helping researchers promote themselves alongside their papers. Helen Eachus is first author on ‘ Glucocorticoid receptor regulates protein chaperone, circadian clock and affective disorder genes in the zebrafish brain’, published in DMM. Helen conducted the research described in this article while a postdoctoral research associate in Dr Vincent Cunliffe's lab at University of Sheffield, Sheffield, UK. She is now a postdoctoral research fellow in the lab of Prof. Soojin Ryu at University of Exeter, Exeter, UK, investigating how stress affects the brain and behaviour in the context of health and disease.


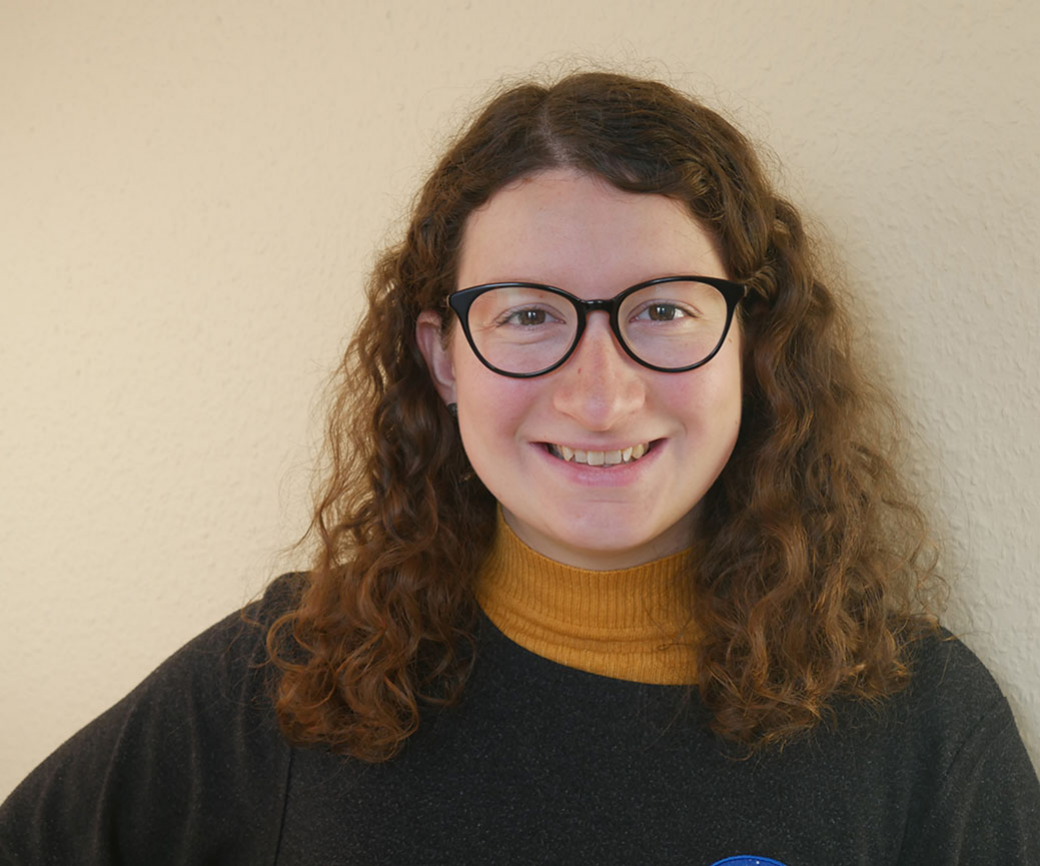


**Helen Eachus**

**How would you explain the main findings of your paper to non-scientific family and friends?**

In this study, we aimed to identify molecules involved in stress signalling in the brain. To address this, we used zebrafish in our experiments because the stress response system is remarkably similar in fish and humans. When under stress, our body produces cortisol, which is our primary stress hormone. Cortisol has wide-ranging effects on the body and brings about an adaptive response to stress. Cortisol does this by binding to its receptor, the glucocorticoid receptor (GR), which is present in most cells in the body, and which regulates the expression of genes to allow us to respond appropriately to stress. Until now, a comprehensive analysis of the genes that are regulated by GR in the brain had not been carried out. Using genetic sequencing technology, we identified hundreds of genes that are regulated by GR in the brain. Many of these genes are involved in protein folding, circadian rhythm and metabolism. Many of the genes are known to be involved in psychiatric disorders, especially anxiety disorders, and we observed anxiety-like behaviours in the GR mutant zebrafish. This suggests that the GR mutant zebrafish might be a promising system to identify molecular mechanisms that underlie anxiety-related behaviours.

[…] the GR mutant zebrafish might be a promising system to identify molecular mechanisms that underlie anxiety-related behaviours.

**What are the potential implications of these results for your field of research?**

Our work identified many previously unknown targets of GR signalling in the brain. This is important, because these targets can now be investigated further, to understand how they might be involved in mediating anxiety behaviours and other stress-related disorders. In the future, further work might be able to develop new treatments for stress-related disorders using the targets identified here.

**What are the main advantages and drawbacks of the experimental system you have used as it relates to the disease you are investigating?**

The zebrafish is arguably the best model system in which to study stress regulation in the brain. This is because the stress response system is very similar in all vertebrates, and zebrafish have the same primary stress hormones as in humans. Around 70% of human genes have zebrafish counterparts, suggesting that the targets we identified are likely to be applicable to humans. The ability of zebrafish to generate large numbers of offspring and the small size of zebrafish larvae mean that the zebrafish is a powerful tool for testing potential new drugs to treat stress-related disorders. One of the drawbacks of using the zebrafish to study the brain is the significant anatomical differences when compared to humans. However, whilst the zebrafish brain is different on an anatomical level, there is a great deal of overlap between fish and higher vertebrates in terms of brain functioning, neurochemistry and molecular conservation of brain circuits.

**Figure:**
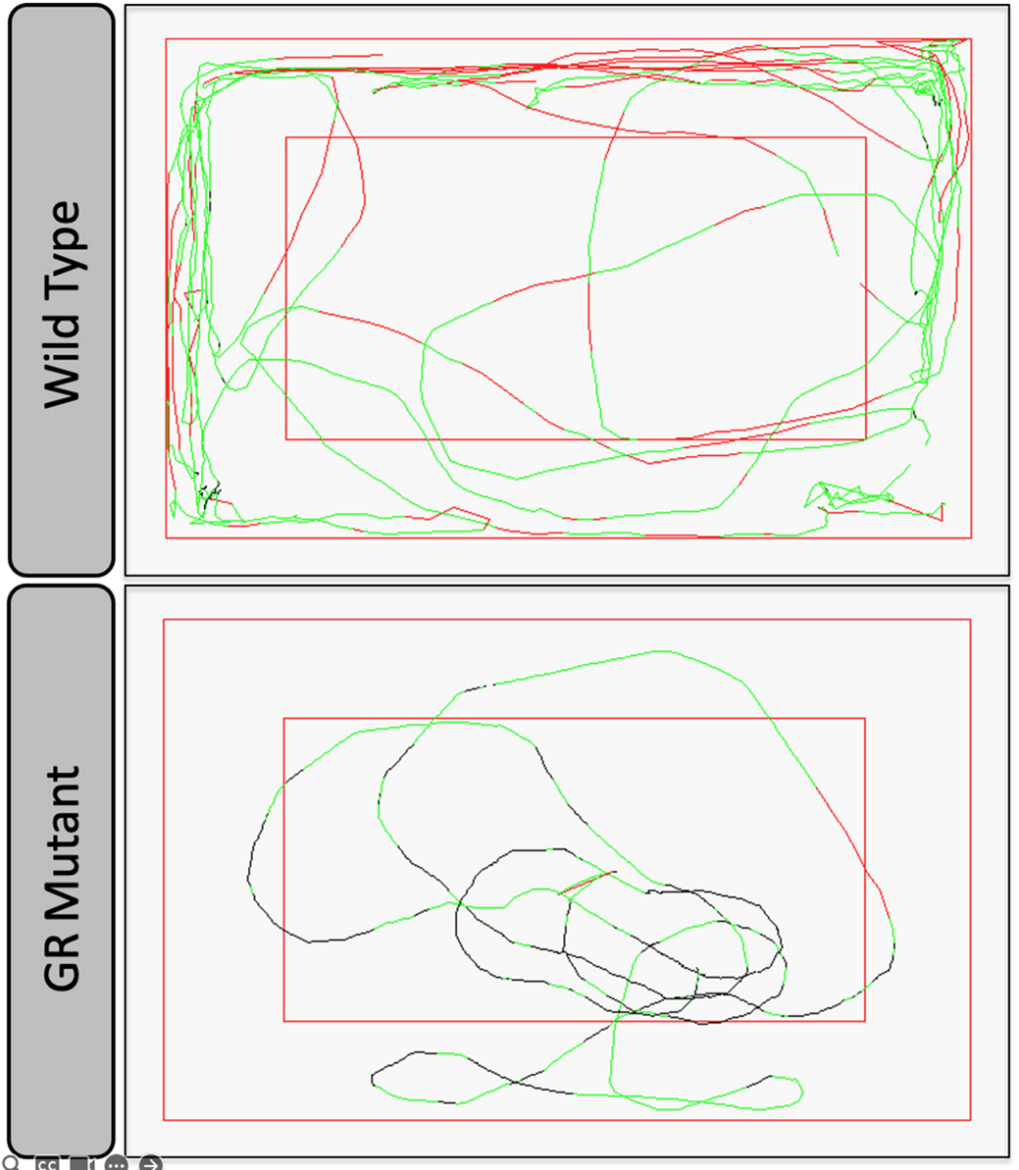
**Tracking of GR mutant and wild-type fish swimming patterns in the open field test in a top-down view of the tank.** We observed striking behavioural differences in the open field test between GR mutant and wild-type zebrafish. GR mutants exhibit freezing behaviour and slow swimming. Red lines indicate fast swimming, green lines indicate medium-speed swimming, and black lines indicate slow swimming.

**What has surprised you the most while conducting your research?**

Whilst conducting this work, I was fascinated by the striking behavioural characteristics I observed in the GR mutant zebrafish. Behaviour is complex and is subject to quite a lot of variation, being influenced by both internal and external factors. For this reason, we often need to measure the behaviour of large numbers of animals to be sure that the observed differences between groups are statistically significant and not down to chance. However, in the GR mutant zebrafish, differences in behaviour compared to the control fish were distinct. For this reason, I was convinced that GR plays a critical role in affecting zebrafish behaviour.

**What do think is the most significant challenge impacting your research at this time and how will this be addressed over the next 10 years?**

We used a ‘bulk’ sequencing approach to identify targets of GR regulation in the whole zebrafish brain. We identified large numbers of GR-regulated genes; however, we used whole-brain samples in our experiment, and the genes we identified might be differentially regulated by GR in different brain regions and different cell types. Therefore, the differences we observed represent an average across the mixture of all cells in the brain, meaning that we may have missed differences that were smaller or region/cell type specific. New technologies including single-cell sequencing and spatially resolved sequencing methods will be able to address this. Currently, these techniques are expensive, and have some significant technical and methodological challenges, but, as the technology improves and becomes more affordable, we will be able to apply these techniques to address important questions regarding the biological embedding of stress in the brain. Ultimately, integration of multi-omic data at different levels and advances in systems bioinformatics will progress translational neuroscience, leading to more effective and efficient biomarker discovery, therapeutic development and mechanistic understanding of brain disorders.

**What changes do you think could improve the professional lives of scientists?**

A major challenge for early-career scientists is navigating the system of short-term contracts. When funding is only available for a few years at a time, this constricts the experimental scope of the project and adds additional pressure and stress to researchers. As we observed in the zebrafish, chronic dysregulation of stress signalling can have significant impact on the brain and behaviour. The stress of wondering whether a research contract will be renewed can contribute to the growing mental-health crisis in academia and impede the ability of researchers to effectively conduct their research. Longer-term funding for projects conducted by researchers at a wider range of career stages would allow researchers to pursue more high-risk (and high-reward) projects, as well as allow them to pursue creative and intellectually driven ideas that may have an impact beyond revenue and rankings. Providing more long-term opportunities would help to lift a weight off the shoulders of early-career researchers, during a time in which they already experience a great deal of pressure.

Providing more long-term opportunities would help to lift a weight off the shoulders of early-career researchers […]

**What's next for you?**

I am fascinated in understanding how stress becomes biologically embedded in the brain, especially in the context of mental health disorders. Using the power of the zebrafish model, my aim is to begin to tackle more of the open questions in the field, such as how stress modulates brain development across the life course. In doing so, I hope to unlock new possibilities in the development of new treatments for mental health disorders, thus improving the lives of the many people who suffer the negative impacts of disorders that are currently poorly understood, and as such have few affective forms of treatment available.
